# High Prevalence of Cardiovascular Disease in End-Stage Kidney Disease Patients Ongoing Hemodialysis in Peru: Why Should We Care About It?

**DOI:** 10.1155/2015/568702

**Published:** 2015-07-29

**Authors:** Katia Bravo-Jaimes, Alvaro Whittembury, Vilma Santivañez

**Affiliations:** ^1^University of Rochester Medical Center, 601 Elmwood Avenue, Box MED, Rochester, NY 14642, USA; ^2^Facultad de Medicina de San Fernando, Universidad Nacional Mayor de San Marcos (UNMSM), Lima, Peru

## Abstract

*Purpose*. To determine clinical, biochemical, and pharmacological characteristics as well as cardiovascular disease prevalence and its associated factors among end-stage kidney disease patients receiving hemodialysis in the main hemodialysis center in Lima, Peru.* Methods*. This cross-sectional study included 103 patients. Clinical charts were reviewed and an echocardiogram was performed to determine prevalence of cardiovascular disease, defined as the presence of systolic/diastolic dysfunction, coronary heart disease, ventricular dysrhythmias, cerebrovascular disease, and/or peripheral vascular disease. Associations between cardiovascular disease and clinical, biochemical, and dialysis factors were sought using prevalence ratio. A robust Poisson regression model was used to quantify possible associations.* Results*. Cardiovascular disease prevalence was 81.6%, mainly due to diastolic dysfunction. It was significantly associated with age older than 50 years, metabolic syndrome, C-reactive protein levels, effective blood flow ≤ 300 mL/min, severe anemia, and absence of mild anemia. However, in the regression analysis only age older than 50 years, effective blood flow ≤ 300 mL/min, and absence of mild anemia were associated.* Conclusions*. Cardiovascular disease prevalence is high in patients receiving hemodialysis in the main center in Lima. Diastolic dysfunction, age, specific hemoglobin levels, and effective blood flow may play an important role.

## 1. Introduction

Chronic kidney disease is a public health problem around the world. It is increasing over time mainly due to an increasing number of diabetic patients, and with nearly 30% of the 170 million diabetic patients eventually developing diabetic nephropathy [1]. Worldwide, most patients with end-stage kidney disease (ESKD) who can access renal replacement therapy are receiving hemodialysis (HD) [2]. In 2013, there were 1,222 patients per million population (pmp) receiving hemodialysis in the USA [2], while, in 2006, there were 280 patients pmp in Latin America [3] and, in 2012, there were 300 patients pmp receiving hemodialysis in Peru [4].

Cardiovascular disease (CVD) is the main cause of death in patients with ESKD [2, 3]. It is estimated that ESKD patients are 5 to 20 times more likely to die because of cardiovascular causes than the general population [5]. Traditional cardiovascular risk factors do not completely explain higher mortality rates among hemodialysis patients [6], and nontraditional risk factors such as anemia, bone mineral disease, hyperhomocysteinemia, inflammation, hypercoagulability, and left ventricular hypertrophy (LVH) [7] have been demonstrated to play an important role in this population. The prevalence of CVD in hemodialysis patients varies across countries; that is, in the USA, ischemic heart disease, heart failure, peripheral vascular disease, and cerebrovascular disease were found in 41% [8], 40% [9], 22% [10], and 13% [11] of these patients, while, in Spain, they were found in 17%, 14%, 6%, and 2% [12] of HD patients.

It is estimated that 300,000 patients have chronic kidney disease in Peru, and more than 9,000 of them require renal replacement therapy [4]. The incidence of ESKD patients receiving hemodialysis is increasing over time [4]; however the prevalence of CVD in these patients remains to be estimated. Thus, this study had the following goals: (1) to determine clinical, biochemical, and pharmacological characteristics among ESKD patients receiving hemodialysis in the main hemodialysis center in Lima, the “Centro Nacional de Salud Renal” (CNSR), (2) to determine CVD prevalence in these patients, and (3) to determine the association between CVD and clinical, biochemical, and dialysis factors in these patients.

## 2. Methods

This cross-sectional study included one hundred and three prevalent HD patients (three sessions of hemodialysis a week for more than three months) from the largest HD center in Lima, Peru (CNSR, EsSalud), using a convenience sampling approach. All patients were adults and had never received renal transplantation or peritoneal dialysis. An informed consent was obtained before enrollment in the study. Ethics Committees from Universidad Nacional Mayor de San Marcos and Centro Nacional de Salud Renal reviewed and approved the study.

Using a standardized form, clinical charts were reviewed to gather the following information: age, gender, prescribed dry weight, cause of ESKD, hypertension status, diabetes status, latest electrocardiogram results, time on hemodialysis, effective blood flow, and current prescription of statins, aspirin, angiotensin-converting enzyme inhibitors (ACE-I), and angiotensin II receptor blockers (ARB). To determine history of CVD (coronary heart disease, cerebrovascular disease, peripheral vascular disease, and ventricular dysrhythmias) and comorbid medical conditions (chronic pulmonary obstructive disease, cancer, dementia, inability to ambulate, peptic ulcer disease, liver disease, HIV status, hepatitis C infection, and hepatitis B infection), chart reviews were supplemented by interviews with the patients, family members, and dialysis staff as necessary.

Patients underwent an evaluation that included (1) anthropometric measurements, (2) blood sample collection, and (3) an echocardiogram. Height and waist circumference were measured after dialysis; the latter was obtained to the nearest centimeter, midway between the lower limit of the rib cage and the iliac crest, with the subject standing, using a flexible and nondistensible tape. Blood samples were obtained before dialysis as part of the routine protocol in the hemodialysis center (a postdialysis sample was used to determine KT/V urea only), without specific instructions to the patients. Blood sample specimens were analyzed for total cholesterol, low-density lipoprotein cholesterol (LDL-C), high-density lipoprotein cholesterol (HDL-C), triglycerides, hemoglobin, calcium, phosphorus, C-reactive protein (CRP), parathyroid hormone, and KT/V urea. A two-dimensional and M-mode echocardiogram was performed using a M2540A model Philips echocardiograph by a single cardiologist at a private clinic using American Society of Echocardiography guidelines for wall thickness and chamber dimensions [13]. The echocardiographic measurements included ejection fraction, left ventricular filling pattern, mitral E/A ratio, and left ventricular mass (calculated from the corrected American Society of Echocardiography method using end diastolic parameters: LV  mass = 0.8{1.04[(IVS + PWT + LVEDD)^3^ − (LVEDD)^3^]} + 0.6).

Charlson comorbidity index and metabolic syndrome status (according to the International Diabetes Federation criteria [14]) were calculated based on the clinical and laboratory results.

Cardiovascular disease (CVD) was defined as the presence of any of the following:

- Coronary heart disease: myocardial infarction or stable angina or unstable angina or coronary artery bypass graft or percutaneous coronary intervention.
- Diastolic dysfunction: abnormal left ventricular filling pattern and/or mitral E/A ratio out of the range 0.7–3.1 if 64 years or under, or 0.5–1.7 if over 64 years [15] found on echocardiogram.
- Systolic dysfunction: ejection fraction less than 55% found on echocardiogram.
- Cerebrovascular disease: atherothrombotic cerebral infarction or carotid endarterectomy or transient ischemic attack.
- Peripheral vascular disease: renal artery stenosis or lower limbs revascularization or any limb amputation.
- Ventricular dysrhythmias: electrocardiographic evidence of ventricular tachycardia or fibrillation.

Data are presented as mean ± SD for continuous variables (median (interquartile range), when not normally distributed) and as fractions for categorical variables. Bivariate analysis searching for associations between CVD and other variables was done using prevalence ratios (PR). Multivariate analysis was performed using a Poisson regression model with robust variance estimation in order to calculate adjusted prevalence ratios (aPR); an enter method was used. Variables included in the multivariate analysis were age, sex, hypertension, metabolic syndrome, CRP levels, severe anemia, absence of mild anemia, and effective blood flow. A *p* value <0.05 was considered statistically significant. All statistical analysis was done using STATA v. 13.0 for Windows.

## 3. Results

### 3.1. Study Patients

A total of 103 patients were included in the study (a third of the population receiving HD at CNSR). Patients were mainly male (*n* = 73, 71%). The mean age was 54.0 ± 14.6 years. The main causes of ESKD were hypertension (30%), glomerulonephritis (22%), and diabetes mellitus (12%). Miscellaneous causes accounted for 30% of the patients (including obstructive uropathy, systemic lupus erythematosus, nephrolithiasis, renal tuberculosis, and polycystic kidney disease). The cause of ESKD could not be determined in 6% of the patients. The mean time on hemodialysis, effective blood flow, and KT/V were 9.7 ± 4.6 years, 326.8 ± 38.1 mL/min, and 1.5 ± 0.3, respectively. The KT/V was less than 1.3 in 9% of patients.

### 3.2. Comorbidities

Ninety-five percent of the patients were hypertensive, 14% were diabetic, 64% had hepatitis C infection, 9% had inability to ambulate, 4% had COPD, 4% had HIV infection, and 1% had hepatitis B infection. The average waist circumference for men and women was 93.0 ± 13.4 cm and 86.0 ± 12.4 cm, respectively. There were 59% of men with waist circumference values above 90 cm and 76% of women with values above 80 cm. Metabolic syndrome was found in 50% of the patients. The average Charlson comorbidity index was 3.5 (range 2–11), and 71% of the patients had an index above 3.

### 3.3. Biochemical Profile

Calcium-phosphorus product above 55 was found in 37% of the patients. Parathyroid hormone levels above 300 pg/mL were found in 54% of the patients. 7.8% of the subjects had albumin levels under 3.6 g/dL. 56% had CRP levels above 0.3 mg/dL. 63% of women had HDL-C level under 50 mg/dL and 41% of men had these levels under 40 mg/dL.

### 3.4. Pharmacological Profile

Three percent of the patients received statins, with 66% of those patients having had a prior cardiovascular event. 60% of patients with coronary heart disease were not prescribed this medication and none of the patients with hypercholesterolemia were prescribed a statin. There were 15% of the subjects receiving aspirin, but just 20% of those with coronary heart disease were prescribed aspirin. ACE-I and ARB were prescribed in 44% and 18% of the patients, respectively.

### 3.5. Echocardiographic Evaluation

Echocardiogram evaluations demonstrated diastolic dysfunction in 75% of the subjects and systolic dysfunction in 10% of them. LVH was noted in 55% of the patients, and amongst these patients 35% were not receiving ACE-I or ARB.

### 3.6. Cardiovascular Disease

CVD was found in 81.6% of the patients (95% CI 73.6–89.5%). Diastolic dysfunction was found in 75% of the subjects, systolic dysfunction in 10%, cerebrovascular disease in 8%, coronary heart disease in 5%, peripheral vascular disease in 1%, and ventricular dysrhythmias in 1%. Clinical and biochemical differences between the patients with and without CVD are shown in Table 1.

### 3.7. CVD Associated Factors

In the bivariate analysis, CVD was significantly associated with age older than 50 years (PR = 1.4; 95% CI 1.1–1.8; *p* = 0.002), metabolic syndrome (PR = 1.2; 95% CI 1.0–1.5; *p* = 0.029), CRP levels (PR = 1.0; 95% CI 1.0-1.1; *p* = 0.015), effective blood flow less than or equal to 300 mL/min (PR = 1.2; 95% CI 1.1–1.3; *p* < 0.001), severe anemia (hemoglobin lesser than 8 g/dL) [16] (PR = 1.2; 95% CI 1.1–1.4; *p* < 0.001) and absence of mild anemia (hemoglobin lesser than 11 g/dL or greater than 11.9 g/dL in females or 12.9 g/dL in males) [16] (PR = 1.6; 95% CI 1.0–2.3; *p* = 0.016). There was a tendency towards significant results for waist circumference greater than 82 cm (PR = 1.3; 95% CI 0.9–1.7; *p* = 0.093). However, the Poisson regression analysis (Table 2) showed that CVD was associated with age older than 50 years (aPR = 1.3; 95% CI 1.1–1.6; *p* = 0.005), effective blood flow less than or equal to 300 mL/min (aPR = 1.2; 95% CI 1.0–1.4; *p* = 0.036), and absence of mild anemia (aPR = 1.5; 95% CI 1.1–2.1; *p* = 0.023).

## 4. Discussion

This study has found an extremely high burden of CVD amongst Peruvian long-term HD patients at CNSR, representing an alarming prevalence that is not substantially different from higher-income countries. Some important associations between CVD disease and clinical, biochemical, and dialysis factors have also been found and potentially represent a challenge for Peruvian nephrologists, especially in the public healthcare system.

CVD prevalence in this study was found to be higher than 80%. When compared to Latin American countries, our sample had higher prevalence of diastolic dysfunction than Argentina (75% versus 40%) [17] and higher prevalence of cerebrovascular disease than Chile (8% versus 7%) [18]. When compared to countries outside Latin America, our sample had higher prevalence of cerebrovascular disease than Spain (8% versus 2%) and lower prevalence of coronary heart disease (5%) and peripheral vascular disease (1%) than Spain (16% and 5%) and the USA (41% and 31.6%) [11, 12].

The main component of CVD in this sample was diastolic dysfunction, which was detected* de novo *in 59% of the patients. This alteration has been recognized as the most common echocardiographic alteration in asymptomatic ESKD patients on HD [19] and is considered a predictor of cardiovascular events in these patients as well [20]. There is evidence suggesting that the severity of diastolic dysfunction and LVH might be ameliorated by adequate correction of anemia and hypertension [21, 22]. For these reasons, it is crucial to detect these problems in a timely fashion, performing an echocardiogram and involving a cardiologist in the multidisciplinary care of patients with CKD [7].

Unfortunately, most of the patients in our sample were not offered an echocardiogram in early stages of kidney disease or at admission to hemodialysis, most likely due to a demand-offer imbalance on the social security health system. The consequences of this disproportion are exemplified by the fact that more than one-third of patients with LVH were not receiving ACE-I or ARB. However there are other situations in which medication prescription status in this cohort was found to be concerning. There was aspirin underuse in patients with coronary heart disease; however specific patient factors, such as increased bleeding risk, may be involved in the low prescription rate and are beyond the scope of this study. Also, statins were not being used in patients with hypercholesterolemia, despite its efficacy to reduce LDL-C levels in this special population [23]. Nevertheless, it is important to note that though these drugs have demonstrated benefit at decreasing stroke incidence and lowering LDL-C levels, respectively, there is no evidence they can reduce mortality in this specific population [1, 2, 24].

As has been previously described in different CKD populations [25–29], we also found that CVD was associated with metabolic syndrome; however this was not confirmed with aPR test. The principal component of this syndrome, abdominal obesity, expressed by waist circumference, has demonstrated to be a predictor of cardiovascular events in ESKD patients [30], and in this study it showed a trend towards significant results. For these reasons we propose that a simple measurement such as waist circumference could potentially denote CVD in this population. However, prospective cohorts are required to evaluate the clinical consequences of these risk factors in Peruvian patients.

The association found between CVD and tight levels of hemoglobin is interesting and thought provoking. Not only patients with severe anemia were found to have higher prevalence of CVD, but also those without mild anemia. This means that patients with hemoglobin levels lower than 11 g/dL or higher than 12.9 g/dL in men or 11.9 g/dL in women had higher prevalence of CVD. These levels are somewhat compatible with those previously reported in the CHOIR study [31], where adverse outcomes were associated with hemoglobin levels higher than 13.0 g/dL. The novel finding in this study is the potential gender-specific difference in hemoglobin targets associated with CVD in this sample. Whether or not this difference is a reflection of the Peruvian population remains to be determined in larger studies. Furthermore, taking into account the varied geography of Peru, further research to determine the appropriate hemoglobin targets in patients with CKD living at high altitude is necessary and will help in determining a possible benefit in adjusting Peruvian Nephrology practice guidelines.

Other associated CVD factors included elevated CRP levels and lower effective blood flow. Elevated CRP levels, defined as above 0.3 mg/dL due to better association with cardiovascular mortality [32], were common in our sample (more than 50%), confirming previous findings in other Peruvian HD cohorts [33]. This demonstrates the underlying inflammatory status that these patients have and could be the resultant of metabolic syndrome. The potential use of CRP as a cardiovascular mortality predictor has been demonstrated in some studies [34] and its use in the Peruvian population should be encouraged. Conversely, effective blood flow seems to be a consequence rather than a predisposing factor in this sample. It might be explained by the fact that patients with CVD may not be able to tolerate elevated blood flows on hemodialysis. Alternatively, another explanation may be that HD techniques mostly applied in Peru use low flux dialyzers, which limits dialysis efficacy.

Unlike other studies where diabetes was the main cause of ESKD [1, 3], the most common cause in this sample was hypertension. This difference in demographics might be explained either by a higher mortality of diabetics at CNSR or that patients with multiple comorbidities receive hemodialysis at other centers better able to care for more complex patients. Another important characteristic of this sample is the high prevalence of hepatitis C virus infection (64%), which is greater than the rates reported by other countries like the USA (5.5–10%) [35], Italy (13.5–31%) [36], France (42%) [37], or Syria (49%) [38], but is comparable to Moldavia (75%) [39]. This may be explained by the long time these patients have lived on hemodialysis (9.7 ± 4.6 years). Filter reuse, dialysate processing, and infection control practices are other possible factors that may explain this increased hepatitis C infection rate [38, 39].

Our study has several limitations, including the sampling approach and sample size which does not allow extrapolating results to the Peruvian population. Additionally, the cross-sectional design does not allow making causal associations. It is important to remark that it was not possible to determine how the CVD prevalence changed with the time under HD, especially considering that for some patients this study provided their first lifetime echocardiogram and thus unrecognized diastolic dysfunction could have not been determined at the time of HD initiation. However several steps were taken to overcome these limitations, including (1) sampling the biggest hemodialysis center in Lima, which has a captive and stable population throughout the years, (2) calculating prevalence ratios instead of odds ratios to not overestimate associations, and (3) including a robust regression analysis to avoid confounding factors.

In conclusion, this study represents the first approach to CVD burden in HD patients in Peru and raises the concern about CVD diagnosis and management in this specific population. Age, effective blood flow, and hemoglobin levels outside a tight target are associated with high prevalence of diastolic dysfunction and CVD in this sample.

## Figures and Tables

**Table 1:** Clinical and biochemical data in the population at CNSR categorized by cardiovascular disease.

	With CVD *N* = 84 (82%)	Without CVD *N* = 19 (18%)	*p* value
Age (years)^*∗*^	56.7 ± 13.6	40.4 ± 11.4	<0.001
Waist circumference (cm)^*∗*^	94.3 ± 13.5	90.0 ± 13.2	0.162
Effective blood flow (mL/min)^*∗*^	321.6 ± 33.1	341.1 ± 36.3	0.006
Total cholesterol (mg/dL)^*∗∗*^	163.5 (49.7)	169.0 (26.5)	0.814
LDL-C (mg/dL)^*∗∗*^	98.0 (33.7)	101.0 (23.5)	0.927
HDL-C (mg/dL)^*∗∗*^	40.0 (9.7)	42.0 (7.5)	0.277
Triglycerides (mg/dL)^*∗∗*^	150.0 (130.5)	136.0 (113.0)	0.652
Calcium (mg/dL)^*∗∗*^	9.5 (2.5)	9.3 (1.6)	0.217
Hemoglobin (mg/dL)^*∗∗*^	12.0 (1.4)	11.4 (5.1)	0.084
CRP (mg/dL)^*∗∗*^	0.5 (0.8)	0.4 (0.4)	0.015
PTH (pg/mL)^*∗∗*^	356.9 (427.3)	475.2 (50.0)	0.235

CNSR: Centro Nacional De Salud Renal; CRP: C-reactive protein; PTH: parathyroid hormone.

^*∗*^Mean ± SD ^*∗∗*^Median (interquartile range).

**Table 2:** Cardiovascular disease: robust Poisson regression model.

Model	aPR	Robust std. err.	*p* value	95% CI
Age older than 50 years	1.3	0.132	0.005	1.1–1.6
EBF ≤ 300	1.2	0.106	0.036	1.0–1.4
Absence of mild anemia	1.5	0.249	0.023	1.1–2.1

Variables included in the initial analysis that did not reach statistical significance were: sex, hypertension, metabolic syndrome, CRP and severe anemia.

## References

[1] Schieppati A., Remuzzi G. (2005). Chronic renal diseases as a public health problem: epidemiology, social, and economic implications. *Kidney International, Supplement*.

[2] U.S.RDS (2013). *USRDS 2013 Annual Data Report: Atlas of Chronic Kidney Disease and End-Stage Renal Disease in the United States*.

[3] Cusumano A. M., Bedat M. C. G., García-García G. (2010). Latin American dialysis and renal transplant registry: 2008 report (data 2006). *Clinical Nephrology*.

[4] Montalvo-Roel I. (2012). *Estado Situacional de los Pacientes con Enfermedad Renal Crónica y la Aplicación de Diálisis como Tratamiento en el Perú*.

[5] Levey A. S., Beto J. A., Coronado B. E. (1998). Controlling the epidemic of cardiovascular disease in chronic renal disease: what do we know? What do we need to learn? Where do we go from here? National Kidney Foundation Task Force on Cardiovascular Disease. *The American Journal of Kidney Diseases*.

[6] Cheung A. K., Sarnak M. J., Yan G. (2000). Atherosclerotic cardiovascular disease risks in chronic hemodialysis patients. *Kidney International*.

[7] K/DOQI Workgroup (2005). K/DOQI clinical practice guidelines for cardiovascular disease in dialysis patients. *American Journal of Kidney Diseases*.

[8] Parfrey P. S., Foley R. N. (1999). The clinical epidemiology of cardiac disease in chronic renal failure. *Journal of the American Society of Nephrology*.

[9] Foley R. N., Parfrey P. S., Sarnak M. J. (1998). Clinical epidemiology of cardiovascular disease in chronic renal disease. *American Journal of Kidney Diseases*.

[10] Sarnak M. J., Levey A. S. (2000). Cardiovascular disease and chronic renal disease: a new paradigm. *American Journal of Kidney Diseases*.

[11] Kundhal K., Lok C. E. (2005). Clinical epidemiology of cardiovascular disease in chronic kidney disease. *Nephron: Clinical Practice*.

[12] Portolés J., López-Gómez J. M., Aljama P., Tato A. M. (2005). Cardiovascular risk in hemodialysis in Spain: prevalence, management and target results (MAR Study). *Nefrologia*.

[13] Park S. H., Shub C., Nobrega T. P., Bailey K. R., Seward J. B. (1996). Two-dimensional echocardiographic calculation of left ventricular mass as recommended by the American Society of Echocardiography: correlation with autopsy and M-mode echocardiography. *Journal of the American Society of Echocardiography*.

[14] Alberti K. G., Zimmet P., Shaw J. (2006). Metabolic syndrome—a new world-wide definition. A consensus statement from the International Diabetes Federation. *Diabetic Medicine*.

[15] Rimington H. (2007). *Echocardiography: A Practical Guide for Reporting*.

[16] WHO (2011). Haemoglobin concentrations for the diagnosis of anaemia and assessment of severity. Vitamin and Mineral Nutrition Information System.

[17] Moretta G., Locatelli A. J., Gadola L. (2008). Rio de La Plata study: a multicenter, cross-sectional study on cardiovascular risk factors and heart failure prevalence in peritoneal dialysis patients in Argentina and Uruguay. *Kidney International. Supplement*.

[18] Sanhueza Villanueva M. E., Cotera A., Elgueta L. (2008). Assessment and follow up of diabetic patients in hemodialysis. *Revista Medica de Chile*.

[19] Glassock R. J., Pecoits-Filho R., Barberato S. H. (2009). Left ventricular mass in chronic kidney disease and ESRD. *Clinical Journal of the American Society of Nephrology*.

[20] Quiroga B., Villaverde M., Abad S., Vega A., Reque J., López-Gómez J. M. (2013). Diastolic dysfunction and high levels of new cardiac biomarkers as risk factors for cardiovascular events and mortality in hemodialysis patients. *Blood Purification*.

[21] Dzgoeva F. U., Gatagonova T. M., Kadzaeva Z. K. (2013). Diastolic dysfunction in different types of left ventricular hypertrophy in patients with end-stage renal failure: impact of long-term erythropoietin therapy. *Terapevticheskii Arkhiv*.

[22] Pecoits-Filho R., Bucharles S., Barberato S. H. (2012). Diastolic heart failure in dialysis patients: mechanisms, diagnostic approach, and treatment. *Seminars in Dialysis*.

[23] Fellström B. C., Jardine A. G., Schmieder R. E. (2009). Rosuvastatin and cardiovascular events in patients undergoing hemodialysis. *The New England Journal of Medicine*.

[24] Nemerovski C. W., Lekura J., Cefaretti M., Mehta P. T., Moore C. L. (2013). Safety and efficacy of statins in patients with end-stage renal disease. *Annals of Pharmacotherapy*.

[25] Bevc S., Potočnik A., Hojs R. (2011). Lipids, waist circumference and body mass index in haemodialysis patients. *Journal of International Medical Research*.

[26] Al Saran K., Elsayed S., Sabry A., Hamada M. (2011). Obesity and metabolic syndrome in hemodialysis patients: single center experience. *Saudi Journal of Kidney Diseases and Transplantation*.

[27] Jalalzadeh M., Mohammadi R., Mirzamohammadi F., Ghadiani M. H. (2011). Prevalence of metabolic syndrome in a hemodialysis population. *Iranian Journal of Kidney Diseases*.

[28] Alfonso A. I. Q., Gallegos R. F., Castillo R. F., Jimenez F. J. G., Rios M. C. G., Garcia I. G. (2015). Study of the metabolic syndrome and obesity in hemodialysis patients. *Nutrición Hospitalaria*.

[29] de José A. P., Verdalles-Guzmán Ú., Abad S. (2014). Metabolic syndrome is associated with cardiovascular events in hemodialysis. *Nefrologia*.

[30] Wu C.-C., Liou H.-H., Su P.-F. (2011). Abdominal obesity is the most significant metabolic syndrome component predictive of cardiovascular events in chronic hemodialysis patients. *Nephrology Dialysis Transplantation*.

[31] Singh A. K., Szczech L., Tang K. L. (2006). Correction of anemia with epoetin alfa in chronic kidney disease. *The New England Journal of Medicine*.

[32] Kawaguchi T., Tong L., Robinson B. M. (2011). C-reactive protein and mortality in hemodialysis patients: the Dialysis Outcomes and Practice Patterns Study (DOPPS). *Nephron—Clinical Practice*.

[33] Valdivia-Mazeyra M. T.-R., Cesar L.-Q., Teresa M. (2012). Asymptomatic cerebrovascular lesions and their relationship to vascular risk factors in patients with end stage renal disease on hemodialysis program. *Revista de la Sociedad Peruana de Medicina Interna*.

[34] Zimmermann J., Herrlinger S., Pruy A., Metzger T., Wanner C. (1999). Inflammation enhances cardiovascular risk and mortality in hemodialysis patients. *Kidney International*.

[35] Finelli L., Miller J. T., Tokars J. I., Alter M. J., Arduino M. J. (2005). National surveillance of dialysis-associated diseases in the United States, 2002. *Seminars in Dialysis*.

[36] Di Napoli A., Pezzotti P., Di Lallo D., Petrosillo N., Trivelloni C., Di Giulio S. (2006). Epidemiology of hepatitis C virus among long-term dialysis patients: a 9-year study in an Italian region. *American Journal of Kidney Diseases*.

[37] Courouce A.-M., Bouchardeau F., Chauveau P. (1995). Hepatitis C virus (HCV) infection in haemodialysed patients: HCV-RNA and anti-HCV antibodies (third-generation assays). *Nephrology Dialysis Transplantation*.

[38] Othman B., Monem F. (2001). Prevalence of antibodies to hepatitis C virus among hemodialysis patients in Damascus, Syria. *Infection*.

[39] Covic A., Iancu L., Apetrei C. (1999). Hepatitis virus infection in haemodialysis patients from Moldavia. *Nephrology Dialysis Transplantation*.

